# (*E*)-1-{4-[Bis(4-meth­oxy­phen­yl)meth­yl]piperazin-1-yl}-3-(4-fluoro­phen­yl)prop-2-en-1-one

**DOI:** 10.1107/S1600536811006210

**Published:** 2011-02-23

**Authors:** Yan-Bo Teng, Zhao-Hui Dai, Bin Wu

**Affiliations:** a School of Pharmacy, Nanjing Medical University, Hanzhong Road No. 140 Nanjing, Nanjing 210029, People’s Republic of China

## Abstract

In the title compound, C_28_H_29_FN_2_O_3_, the conformation about the ethene bond is *E*. The piperazine ring adopts a chair conformation. In the crystal, mol­ecules are linked by inter­molecular C—H⋯O hydrogen bonds.

## Related literature

For properties of cinnamic acid derivatives, see: Shi *et al.* (2005 ▶); Point *et al.* (1998 ▶). For synthetic procedures, see: Wu *et al.* (2008 ▶). For a related structure, see: Mouillé *et al.* (1975 ▶).
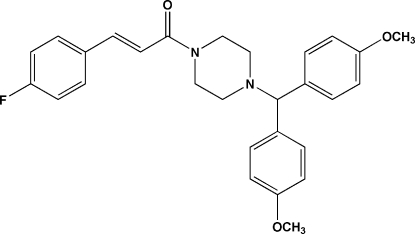


## Experimental

### 

#### Crystal data

C_28_H_29_FN_2_O_3_
*M* *_r_* = 460.53
Monoclinic, 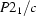

*a* = 10.235 (2) Å
*b* = 7.8420 (16) Å
*c* = 30.385 (6) Å
β = 96.65 (3)°
*V* = 2422.4 (8) Å^3^
*Z* = 4
Mo *K*α radiation
μ = 0.09 mm^−1^
*T* = 293 K
0.20 × 0.10 × 0.10 mm

#### Data collection

Enraf–Nonius CAD-4 diffractometer
Absorption correction: ψ scan (North *et al.*, 1968) ▶ *T*_min_ = 0.983, *T*_max_ = 0.991
4730 measured reflections
4463 independent reflections
2366 reflections with *I* > 2σ(*I*)
*R*_int_ = 0.031
3 standard reflections every 200 reflections  intensity decay: 1%

#### Refinement

*R*[*F* ^2^ > 2σ(*F* ^2^)] = 0.061
*wR*(*F* ^2^) = 0.170
*S* = 1.01
4463 reflections
307 parameters
H-atom parameters constrained
Δρ_max_ = 0.13 e Å^−3^
Δρ_min_ = −0.18 e Å^−3^

### 

Data collection: *CAD-4 EXPRESS* (Enraf–Nonius, 1994 ▶); cell refinement: *CAD-4 EXPRESS*; data reduction: *XCAD4* (Harms & Wocadlo, 1995 ▶); program(s) used to solve structure: *SHELXS97* (Sheldrick, 2008 ▶); program(s) used to refine structure: *SHELXL97* (Sheldrick, 2008 ▶); molecular graphics: *SHELXTL* (Sheldrick, 2008 ▶); software used to prepare material for publication: *SHELXTL*.

## Supplementary Material

Crystal structure: contains datablocks I, global. DOI: 10.1107/S1600536811006210/pv2381sup1.cif

Structure factors: contains datablocks I. DOI: 10.1107/S1600536811006210/pv2381Isup2.hkl

Additional supplementary materials:  crystallographic information; 3D view; checkCIF report

## Figures and Tables

**Table 1:** Hydrogen-bond geometry (Å, °)

*D*—H⋯*A*	*D*—H	H⋯*A*	*D*⋯*A*	*D*—H⋯*A*
C5—H5*A*⋯O1^i^	0.93	2.28	3.131 (4)	152
C17—H17*A*⋯O3^ii^	0.93	2.60	3.499 (4)	163

Symmetry codes: (i) 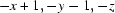; (ii) 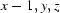.

**Fig. 1.**
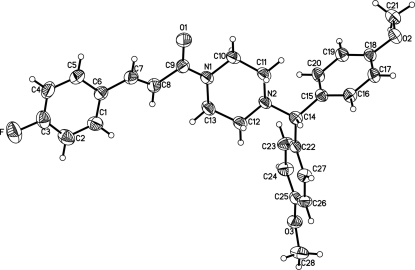
Molecular structure of the title compound, showing the atom labeling scheme and 70% probability displacement ellipsoids.

**Fig. 2.**
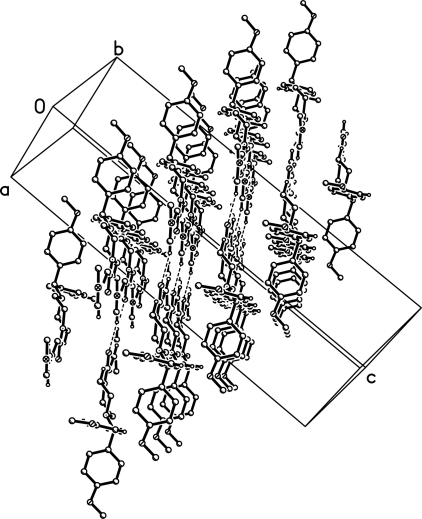
Packing diagram of the title compound showing hydrogen bonds as dashed lines.

## supplementary crystallographic information

### Comment

Cinnamic acid derivatives have been reported to possess many useful properties, including alpha-glucosidase inhibition, acyl-CoA inhibition, LDL-oxidation inhibition, tyrosinase inhibition, antioxidant, antimicrobial, neuroprotective activities (Shi *et al.*, 2005; Point *et al.*, 1998). We report here the synthesis and crystal structure of a novel cinnamic acid derivative.

In the title molecule (Fig. 1), the conformation about the ethene bond C7═C8 is E. The piperazine ring adopts a chair conformation. There are intramolecular and intermolecular C—H···O hydrogen bonds in the title compound (Fig. 2) which consilidate the crystal structure. The bond lenths and angles in the title compound agree well with the corresponding bond lengths and angles in a closely related compound, *trans*-cinnamyl-1-diphenylmethyl-4-piperazine (Mouillé *et al.*, 1975).

### Experimental

The synthesis follows the method of Wu *et al.* (2008). A mixture of (*E*)-3-(4-fluoro phenyl)acrylic acid (1.66 g; 10 mmol), dimethyl sulfoxide (4 ml) and dichloromethane (60 ml) was stirred for 6 h at room temperature. The solvent was removed under reduced pressure. The residue was dissolved in acetone (60 ml) and reacted with 1-(bis(4-methoxyphenyl)methyl) piperazine (4.69 g; 15 mmol) in the presence of triethylamine (12 ml) for 5 h at room temperature. The resultant mixture was cooled. The solid thus obtained was filtered and recrystallized from ethanol to afford the title compound. Pale-yellow single crystals of the title compound suitable for *X*-ray diffraction studies were grown from a mixture of CHCl_3_ and hexane (1:1) by slow evaporation at room temperature.

### Refinement

All H atoms were placed geometrically at distances C—H = 0.93, 0.96, 0.97 and 0.98 Å for aryl, methyl, methylene and methyne type H-atoms, respectively, and included in the refinement in riding motion approximation with *U*_iso_(H) = 1.2 or 1.5*U*_eq_ of the carrier atom.

### Crystal data

**Table:**

C_28_H_29_FN_2_O_3_	*F*(000) = 976
*M**_r_* = 460.53	*D*_x_ = 1.263 Mg m^−^^3^
Monoclinic, *P*2_1_/*c*	Mo *K*α radiation, λ = 0.71073 Å
Hall symbol: -P 2ybc	Cell parameters from 25 reflections
*a* = 10.235 (2) Å	θ = 10–13°
*b* = 7.8420 (16) Å	µ = 0.09 mm^−^^1^
*c* = 30.385 (6) Å	*T* = 293 K
β = 96.65 (3)°	Block, pale-yellow
*V* = 2422.4 (8) Å^3^	0.20 × 0.10 × 0.10 mm
*Z* = 4	

### Data collection

**Table:**

Enraf–Nonius CAD-4 diffractometer	2366 reflections with *I* > 2σ(*I*)
Radiation source: fine-focus sealed tube	*R*_int_ = 0.031
graphite	θ_max_ = 25.4°, θ_min_ = 1.4°
ω and 2θ scans	*h* = 0→12
Absorption correction: multi-scan ψ scan	*k* = 0→9
*T*_min_ = 0.983, *T*_max_ = 0.991	*l* = −36→36
4730 measured reflections	3 standard reflections every 200 reflections
4463 independent reflections	intensity decay: 1%

### Refinement

**Table:**

Refinement on *F*^2^	Primary atom site location: structure-invariant direct methods
Least-squares matrix: full	Secondary atom site location: difference Fourier map
*R*[*F*^2^ > 2σ(*F*^2^)] = 0.061	Hydrogen site location: inferred from neighbouring sites
*wR*(*F*^2^) = 0.170	H-atom parameters constrained
*S* = 1.01	*w* = 1/[σ^2^(*F*_o_^2^) + (0.080*P*)^2^] where *P* = (*F*_o_^2^ + 2*F*_c_^2^)/3
4463 reflections	(Δ/σ)_max_ < 0.001
307 parameters	Δρ_max_ = 0.13 e Å^−^^3^
0 restraints	Δρ_min_ = −0.18 e Å^−^^3^

### Special details

**Table:**

Experimental. (North *et al.*, 1968)
Geometry. All e.s.d.'s (except the e.s.d. in the dihedral angle between two l.s. planes) are estimated using the full covariance matrix. The cell e.s.d.'s are taken into account individually in the estimation of e.s.d.'s in distances, angles and torsion angles; correlations between e.s.d.'s in cell parameters are only used when they are defined by crystal symmetry. An approximate (isotropic) treatment of cell e.s.d.'s is used for estimating e.s.d.'s involving l.s. planes.
Refinement. Refinement of *F*^2^ against ALL reflections. The weighted *R*-factor *wR* and goodness of fit *S* are based on *F*^2^, conventional *R*-factors *R* are based on *F*, with *F* set to zero for negative *F*^2^. The threshold expression of *F*^2^ > σ(*F*^2^) is used only for calculating *R*-factors(gt) *etc*. and is not relevant to the choice of reflections for refinement. *R*-factors based on *F*^2^ are statistically about twice as large as those based on *F*, and *R*- factors based on ALL data will be even larger.

### Fractional atomic coordinates and isotropic or equivalent isotropic displacement parameters (Å^2^)

**Table:**

	*x*	*y*	*z*	*U*_iso_*/*U*_eq_	
F	1.1036 (2)	−0.8047 (3)	0.03071 (8)	0.1138 (8)	
N1	0.5564 (2)	0.0310 (3)	0.08123 (8)	0.0606 (7)	
O1	0.4552 (2)	−0.1690 (3)	0.03651 (7)	0.0783 (7)	
C1	0.9101 (3)	−0.4669 (4)	0.06924 (11)	0.0723 (10)	
H1A	0.9196	−0.3770	0.0893	0.087*	
O2	0.06003 (19)	0.8563 (3)	0.14269 (7)	0.0658 (6)	
N2	0.5216 (2)	0.3082 (3)	0.13971 (8)	0.0540 (6)	
C2	1.0149 (4)	−0.5726 (5)	0.06570 (13)	0.0838 (11)	
H2A	1.0948	−0.5563	0.0831	0.101*	
O3	0.93784 (18)	0.7764 (3)	0.25379 (7)	0.0653 (6)	
C3	0.9982 (4)	−0.7018 (5)	0.03591 (13)	0.0779 (10)	
C4	0.8809 (4)	−0.7385 (5)	0.01176 (12)	0.0841 (11)	
H4A	0.8712	−0.8333	−0.0067	0.101*	
C5	0.7775 (4)	−0.6302 (5)	0.01579 (11)	0.0755 (10)	
H5A	0.6967	−0.6512	−0.0007	0.091*	
C6	0.7909 (3)	−0.4905 (4)	0.04381 (10)	0.0592 (8)	
C7	0.6790 (3)	−0.3740 (4)	0.04508 (10)	0.0636 (9)	
H7A	0.5982	−0.4155	0.0324	0.076*	
C8	0.6773 (3)	−0.2193 (4)	0.06162 (10)	0.0619 (9)	
H8A	0.7551	−0.1731	0.0755	0.074*	
C9	0.5553 (3)	−0.1162 (4)	0.05889 (10)	0.0574 (8)	
C10	0.4434 (3)	0.1440 (4)	0.07401 (10)	0.0677 (9)	
H10A	0.3688	0.0814	0.0595	0.081*	
H10B	0.4629	0.2365	0.0546	0.081*	
C11	0.4086 (3)	0.2162 (4)	0.11695 (10)	0.0633 (9)	
H11A	0.3345	0.2932	0.1112	0.076*	
H11B	0.3834	0.1246	0.1357	0.076*	
C12	0.6265 (3)	0.1835 (4)	0.15010 (11)	0.0662 (9)	
H12A	0.5962	0.0944	0.1686	0.079*	
H12B	0.7016	0.2387	0.1666	0.079*	
C13	0.6676 (3)	0.1057 (4)	0.10871 (11)	0.0658 (9)	
H13A	0.7075	0.1927	0.0919	0.079*	
H13B	0.7331	0.0182	0.1167	0.079*	
C14	0.4907 (3)	0.4005 (4)	0.17929 (9)	0.0539 (8)	
H14A	0.4654	0.3170	0.2008	0.065*	
C15	0.3763 (3)	0.5219 (4)	0.16789 (10)	0.0496 (7)	
C16	0.2791 (3)	0.5383 (4)	0.19576 (10)	0.0562 (8)	
H16A	0.2837	0.4723	0.2213	0.067*	
C17	0.1757 (3)	0.6503 (4)	0.18645 (10)	0.0588 (8)	
H17A	0.1113	0.6587	0.2056	0.071*	
C18	0.1677 (3)	0.7490 (4)	0.14916 (10)	0.0517 (7)	
C19	0.2627 (3)	0.7373 (4)	0.12093 (10)	0.0564 (8)	
H19A	0.2579	0.8045	0.0956	0.068*	
C20	0.3658 (3)	0.6239 (4)	0.13073 (10)	0.0576 (8)	
H20A	0.4302	0.6165	0.1116	0.069*	
C21	0.0545 (3)	0.9718 (4)	0.10672 (11)	0.0739 (10)	
H21A	−0.0246	1.0382	0.1056	0.111*	
H21B	0.0548	0.9093	0.0796	0.111*	
H21C	0.1295	1.0461	0.1106	0.111*	
C22	0.6118 (3)	0.4964 (4)	0.20031 (9)	0.0487 (7)	
C23	0.6794 (3)	0.6068 (4)	0.17551 (10)	0.0654 (9)	
H23A	0.6512	0.6209	0.1455	0.078*	
C24	0.7869 (3)	0.6956 (4)	0.19426 (10)	0.0640 (9)	
H24A	0.8311	0.7679	0.1768	0.077*	
C25	0.8303 (3)	0.6793 (4)	0.23854 (10)	0.0516 (7)	
C26	0.7663 (3)	0.5699 (4)	0.26369 (10)	0.0594 (8)	
H26A	0.7956	0.5555	0.2936	0.071*	
C27	0.6569 (3)	0.4799 (4)	0.24439 (10)	0.0583 (8)	
H27A	0.6134	0.4066	0.2619	0.070*	
C28	0.9994 (3)	0.7437 (5)	0.29707 (11)	0.0853 (11)	
H28A	1.0722	0.8203	0.3037	0.128*	
H28B	1.0305	0.6282	0.2989	0.128*	
H28C	0.9372	0.7606	0.3180	0.128*	

### Atomic displacement parameters (Å^2^)

**Table:**

	*U*^11^	*U*^22^	*U*^33^	*U*^12^	*U*^13^	*U*^23^
F	0.1173 (18)	0.1002 (17)	0.1298 (19)	0.0444 (15)	0.0391 (15)	0.0195 (15)
N1	0.0469 (15)	0.0636 (17)	0.0693 (17)	0.0060 (14)	−0.0021 (13)	−0.0110 (15)
O1	0.0608 (14)	0.0883 (17)	0.0834 (16)	−0.0077 (13)	−0.0015 (12)	−0.0212 (13)
C1	0.075 (2)	0.053 (2)	0.084 (2)	0.0013 (19)	−0.0119 (19)	−0.0117 (18)
O2	0.0542 (12)	0.0670 (14)	0.0768 (15)	0.0124 (12)	0.0106 (10)	0.0043 (12)
N2	0.0361 (13)	0.0562 (15)	0.0682 (16)	0.0014 (12)	−0.0003 (11)	−0.0075 (13)
C2	0.075 (2)	0.059 (2)	0.113 (3)	0.006 (2)	−0.010 (2)	−0.003 (2)
O3	0.0502 (12)	0.0750 (15)	0.0689 (14)	−0.0098 (12)	−0.0008 (10)	−0.0058 (12)
C3	0.087 (3)	0.067 (3)	0.083 (3)	0.019 (2)	0.026 (2)	0.017 (2)
C4	0.109 (3)	0.079 (3)	0.066 (2)	0.015 (3)	0.017 (2)	−0.018 (2)
C5	0.081 (2)	0.080 (3)	0.065 (2)	−0.004 (2)	0.0024 (18)	−0.019 (2)
C6	0.067 (2)	0.0534 (19)	0.0571 (18)	−0.0045 (17)	0.0063 (16)	−0.0043 (16)
C7	0.059 (2)	0.069 (2)	0.062 (2)	−0.0046 (18)	0.0049 (16)	−0.0068 (18)
C8	0.0550 (19)	0.060 (2)	0.070 (2)	−0.0064 (17)	0.0063 (15)	−0.0123 (18)
C9	0.0530 (19)	0.063 (2)	0.0568 (19)	−0.0047 (17)	0.0098 (15)	−0.0026 (17)
C10	0.0502 (18)	0.075 (2)	0.074 (2)	0.0085 (18)	−0.0097 (16)	−0.0102 (18)
C11	0.0391 (16)	0.067 (2)	0.081 (2)	0.0023 (16)	−0.0036 (15)	−0.0091 (18)
C12	0.0475 (18)	0.063 (2)	0.085 (2)	0.0043 (16)	−0.0082 (16)	−0.0163 (18)
C13	0.0429 (17)	0.064 (2)	0.089 (2)	0.0022 (16)	0.0026 (16)	−0.0136 (19)
C14	0.0489 (17)	0.0533 (18)	0.0598 (19)	−0.0024 (15)	0.0077 (14)	0.0045 (16)
C15	0.0384 (15)	0.0494 (17)	0.0606 (18)	−0.0038 (14)	0.0045 (13)	−0.0015 (15)
C16	0.0540 (18)	0.0579 (19)	0.0587 (18)	−0.0039 (16)	0.0150 (15)	0.0081 (16)
C17	0.0470 (17)	0.065 (2)	0.066 (2)	0.0046 (16)	0.0166 (15)	0.0020 (17)
C18	0.0414 (16)	0.0525 (18)	0.0610 (19)	−0.0016 (15)	0.0059 (14)	−0.0063 (16)
C19	0.0553 (18)	0.0559 (19)	0.0588 (19)	0.0031 (16)	0.0096 (15)	0.0058 (16)
C20	0.0430 (16)	0.067 (2)	0.066 (2)	0.0037 (16)	0.0170 (14)	0.0057 (17)
C21	0.059 (2)	0.074 (2)	0.088 (3)	0.0111 (18)	0.0009 (18)	0.006 (2)
C22	0.0414 (15)	0.0493 (17)	0.0557 (18)	0.0038 (14)	0.0063 (13)	−0.0022 (15)
C23	0.062 (2)	0.085 (2)	0.0485 (18)	−0.0180 (19)	0.0023 (15)	0.0081 (17)
C24	0.0528 (18)	0.079 (2)	0.061 (2)	−0.0176 (18)	0.0077 (15)	0.0101 (18)
C25	0.0406 (16)	0.0512 (18)	0.063 (2)	0.0044 (15)	0.0061 (15)	−0.0050 (16)
C26	0.0550 (18)	0.073 (2)	0.0476 (17)	0.0007 (18)	−0.0041 (15)	0.0037 (16)
C27	0.0533 (18)	0.062 (2)	0.0598 (19)	−0.0049 (17)	0.0067 (15)	0.0113 (17)
C28	0.063 (2)	0.100 (3)	0.086 (3)	−0.003 (2)	−0.0217 (19)	0.003 (2)

### Geometric parameters (Å, °)

**Table:**

F—C3	1.370 (4)	C12—H12A	0.9700
N1—C9	1.338 (4)	C12—H12B	0.9700
N1—C10	1.453 (4)	C13—H13A	0.9700
N1—C13	1.455 (4)	C13—H13B	0.9700
O1—C9	1.235 (3)	C14—C15	1.517 (4)
C1—C2	1.370 (4)	C14—C22	1.525 (4)
C1—C6	1.379 (4)	C14—H14A	0.9800
C1—H1A	0.9300	C15—C20	1.378 (4)
O2—C18	1.382 (3)	C15—C16	1.385 (4)
O2—C21	1.416 (3)	C16—C17	1.380 (4)
N2—C12	1.459 (3)	C16—H16A	0.9300
N2—C11	1.466 (3)	C17—C18	1.367 (4)
N2—C14	1.469 (3)	C17—H17A	0.9300
C2—C3	1.357 (5)	C18—C19	1.372 (4)
C2—H2A	0.9300	C19—C20	1.386 (4)
O3—C25	1.374 (3)	C19—H19A	0.9300
O3—C28	1.414 (3)	C20—H20A	0.9300
C3—C4	1.364 (5)	C21—H21A	0.9600
C4—C5	1.373 (5)	C21—H21B	0.9600
C4—H4A	0.9300	C21—H21C	0.9600
C5—C6	1.385 (4)	C22—C27	1.372 (4)
C5—H5A	0.9300	C22—C23	1.384 (4)
C6—C7	1.469 (4)	C23—C24	1.370 (4)
C7—C8	1.314 (4)	C23—H23A	0.9300
C7—H7A	0.9300	C24—C25	1.373 (4)
C8—C9	1.482 (4)	C24—H24A	0.9300
C8—H8A	0.9300	C25—C26	1.365 (4)
C10—C11	1.503 (4)	C26—C27	1.394 (4)
C10—H10A	0.9700	C26—H26A	0.9300
C10—H10B	0.9700	C27—H27A	0.9300
C11—H11A	0.9700	C28—H28A	0.9600
C11—H11B	0.9700	C28—H28B	0.9600
C12—C13	1.501 (4)	C28—H28C	0.9600
			
C9—N1—C10	119.3 (2)	C12—C13—H13B	109.3
C9—N1—C13	126.8 (3)	H13A—C13—H13B	108.0
C10—N1—C13	113.4 (2)	N2—C14—C15	110.8 (2)
C2—C1—C6	121.7 (3)	N2—C14—C22	110.1 (2)
C2—C1—H1A	119.2	C15—C14—C22	110.8 (2)
C6—C1—H1A	119.2	N2—C14—H14A	108.3
C18—O2—C21	117.3 (2)	C15—C14—H14A	108.3
C12—N2—C11	107.0 (2)	C22—C14—H14A	108.3
C12—N2—C14	112.1 (2)	C20—C15—C16	117.0 (3)
C11—N2—C14	113.3 (2)	C20—C15—C14	122.4 (3)
C3—C2—C1	117.7 (3)	C16—C15—C14	120.6 (3)
C3—C2—H2A	121.1	C17—C16—C15	121.5 (3)
C1—C2—H2A	121.1	C17—C16—H16A	119.3
C25—O3—C28	117.8 (3)	C15—C16—H16A	119.3
C2—C3—C4	123.5 (4)	C18—C17—C16	120.1 (3)
C2—C3—F	118.5 (4)	C18—C17—H17A	120.0
C4—C3—F	118.0 (4)	C16—C17—H17A	120.0
C3—C4—C5	117.5 (3)	C17—C18—C19	120.1 (3)
C3—C4—H4A	121.3	C17—C18—O2	115.6 (3)
C5—C4—H4A	121.3	C19—C18—O2	124.3 (3)
C4—C5—C6	121.5 (3)	C18—C19—C20	119.1 (3)
C4—C5—H5A	119.3	C18—C19—H19A	120.5
C6—C5—H5A	119.3	C20—C19—H19A	120.5
C1—C6—C5	118.0 (3)	C15—C20—C19	122.3 (3)
C1—C6—C7	122.9 (3)	C15—C20—H20A	118.9
C5—C6—C7	119.1 (3)	C19—C20—H20A	118.9
C8—C7—C6	129.0 (3)	O2—C21—H21A	109.5
C8—C7—H7A	115.5	O2—C21—H21B	109.5
C6—C7—H7A	115.5	H21A—C21—H21B	109.5
C7—C8—C9	122.1 (3)	O2—C21—H21C	109.5
C7—C8—H8A	119.0	H21A—C21—H21C	109.5
C9—C8—H8A	119.0	H21B—C21—H21C	109.5
O1—C9—N1	121.8 (3)	C27—C22—C23	117.2 (3)
O1—C9—C8	119.2 (3)	C27—C22—C14	121.9 (3)
N1—C9—C8	119.0 (3)	C23—C22—C14	120.9 (3)
N1—C10—C11	111.3 (2)	C24—C23—C22	121.3 (3)
N1—C10—H10A	109.4	C24—C23—H23A	119.3
C11—C10—H10A	109.4	C22—C23—H23A	119.3
N1—C10—H10B	109.4	C23—C24—C25	120.9 (3)
C11—C10—H10B	109.4	C23—C24—H24A	119.6
H10A—C10—H10B	108.0	C25—C24—H24A	119.6
N2—C11—C10	110.0 (2)	C26—C25—C24	119.0 (3)
N2—C11—H11A	109.7	C26—C25—O3	125.2 (3)
C10—C11—H11A	109.7	C24—C25—O3	115.8 (3)
N2—C11—H11B	109.7	C25—C26—C27	119.8 (3)
C10—C11—H11B	109.7	C25—C26—H26A	120.1
H11A—C11—H11B	108.2	C27—C26—H26A	120.1
N2—C12—C13	111.2 (3)	C22—C27—C26	121.8 (3)
N2—C12—H12A	109.4	C22—C27—H27A	119.1
C13—C12—H12A	109.4	C26—C27—H27A	119.1
N2—C12—H12B	109.4	O3—C28—H28A	109.5
C13—C12—H12B	109.4	O3—C28—H28B	109.5
H12A—C12—H12B	108.0	H28A—C28—H28B	109.5
N1—C13—C12	111.6 (2)	O3—C28—H28C	109.5
N1—C13—H13A	109.3	H28A—C28—H28C	109.5
C12—C13—H13A	109.3	H28B—C28—H28C	109.5
N1—C13—H13B	109.3		
			
C6—C1—C2—C3	0.4 (5)	N2—C14—C15—C20	−43.6 (4)
C1—C2—C3—C4	−4.6 (6)	C22—C14—C15—C20	79.0 (3)
C1—C2—C3—F	177.5 (3)	N2—C14—C15—C16	138.6 (3)
C2—C3—C4—C5	4.9 (6)	C22—C14—C15—C16	−98.9 (3)
F—C3—C4—C5	−177.2 (3)	C20—C15—C16—C17	0.7 (4)
C3—C4—C5—C6	−1.0 (5)	C14—C15—C16—C17	178.7 (3)
C2—C1—C6—C5	3.1 (5)	C15—C16—C17—C18	−0.4 (4)
C2—C1—C6—C7	−176.5 (3)	C16—C17—C18—C19	−0.1 (4)
C4—C5—C6—C1	−2.8 (5)	C16—C17—C18—O2	179.6 (2)
C4—C5—C6—C7	176.9 (3)	C21—O2—C18—C17	174.3 (3)
C1—C6—C7—C8	15.0 (5)	C21—O2—C18—C19	−5.9 (4)
C5—C6—C7—C8	−164.7 (3)	C17—C18—C19—C20	0.2 (4)
C6—C7—C8—C9	178.1 (3)	O2—C18—C19—C20	−179.5 (3)
C10—N1—C9—O1	−9.2 (4)	C16—C15—C20—C19	−0.6 (4)
C13—N1—C9—O1	179.7 (3)	C14—C15—C20—C19	−178.6 (3)
C10—N1—C9—C8	171.6 (3)	C18—C19—C20—C15	0.2 (4)
C13—N1—C9—C8	0.6 (5)	N2—C14—C22—C27	−128.0 (3)
C7—C8—C9—O1	−7.7 (5)	C15—C14—C22—C27	109.0 (3)
C7—C8—C9—N1	171.5 (3)	N2—C14—C22—C23	53.2 (4)
C9—N1—C10—C11	137.5 (3)	C15—C14—C22—C23	−69.8 (3)
C13—N1—C10—C11	−50.3 (4)	C27—C22—C23—C24	0.0 (5)
C12—N2—C11—C10	−63.1 (3)	C14—C22—C23—C24	178.9 (3)
C14—N2—C11—C10	172.9 (2)	C22—C23—C24—C25	−0.7 (5)
N1—C10—C11—N2	57.9 (3)	C23—C24—C25—C26	1.4 (5)
C11—N2—C12—C13	62.0 (3)	C23—C24—C25—O3	−179.1 (3)
C14—N2—C12—C13	−173.2 (2)	C28—O3—C25—C26	9.6 (4)
C9—N1—C13—C12	−139.8 (3)	C28—O3—C25—C24	−169.8 (3)
C10—N1—C13—C12	48.7 (4)	C24—C25—C26—C27	−1.4 (4)
N2—C12—C13—N1	−55.2 (4)	O3—C25—C26—C27	179.2 (3)
C12—N2—C14—C15	−176.8 (2)	C23—C22—C27—C26	0.0 (4)
C11—N2—C14—C15	−55.6 (3)	C14—C22—C27—C26	−178.9 (3)
C12—N2—C14—C22	60.3 (3)	C25—C26—C27—C22	0.7 (5)
C11—N2—C14—C22	−178.5 (2)		

### Hydrogen-bond geometry (Å, °)

**Table:**

*D*—H···*A*	*D*—H	H···*A*	*D*···*A*	*D*—H···*A*
C5—H5A···O1^i^	0.93	2.28	3.131 (4)	152
C17—H17A···O3^ii^	0.93	2.60	3.499 (4)	163
C10—H10A···O1	0.97	2.30	2.715 (4)	105
C7—H7A···O1	0.93	2.43	2.786 (4)	102

Symmetry codes: (i) −*x*+1, −*y*−1, −*z*; (ii) *x*−1, *y*, *z*.
